# Toll-Like Receptors in the Pathogenesis of Essential Hypertension. A Forthcoming Immune-Driven Theory in Full Effect

**DOI:** 10.3390/ijms22073451

**Published:** 2021-03-26

**Authors:** Antonios Lazaridis, Eleni Gavriilaki, Stella Douma, Eugenia Gkaliagkousi

**Affiliations:** 1 3rd Department of Internal Medicine, Papageorgiou Hospital, Aristotle University of Thessaloniki, 56403 Thessaloniki, Greece; spanbiol@hotmail.com (A.L.); sdouma@auth.gr (S.D.); eugalant@yahoo.com (E.G.); 2 Hematology Department, Bone Marrow Transplantation Unit, G. Papanicolaou Hospital, 57010 Thessaloniki, Greece

**Keywords:** hypertension, innate immunity, toll-like receptors, inflammation, cytokines

## Abstract

Essential hypertension (EH) is a highly heterogenous disease with a complex etiology. Recent evidence highlights the significant contribution of subclinical inflammation, triggered and sustained by excessive innate immune system activation in the pathogenesis of the disease. Toll-like receptors (TLRs) have been implied as novel effectors in this inflammatory environment since they can significantly stimulate the production of pro-inflammatory cytokines, the migration and proliferation of smooth muscle cells and the generation of reactive oxygen species (ROS), facilitating a low-intensity inflammatory background that is evident from the very early stages of hypertension. Furthermore, the net result of their activation is oxidative stress, endothelial dysfunction, vascular remodeling, and finally, vascular target organ damage, which forms the pathogenetic basis of EH. Importantly, evidence of augmented TLR expression and activation in hypertension has been documented not only in immune but also in several non-immune cells located in the central nervous system, the kidneys, and the vasculature which form the pathogenetic core systems operating in hypertensive disease. In this review, we will try to highlight the contribution of innate immunity in the pathogenesis of hypertension by clarifying the deleterious role of TLR signaling in promoting inflammation and facilitating hypertensive vascular damage.

## 1. Introduction

Essential hypertension (EH) has an independent and continuous relationship with the incidence of several cardiovascular events, and it is a leading cause of cardiovascular morbidity and mortality, surpassing all other cardiovascular risk factors such as diabetes mellitus, smoking, and hyperlipidemia [1,2]. In particular, the global burden of EH currently affects almost 30 to 45% of all adults and accounts for almost 10 million deaths and over 200 million disability-adjusted life years worldwide, with a constantly increasing tendency [3,4]. The continuous and close relationship of hypertension with cardiovascular disease, along with its overall prevalence worldwide, clearly render EH a major issue of public health that calls for immediate action.

Despite advances in awareness and pharmacotherapy, no unifying mechanism, and thus, no single therapeutic target exists, rendering control rates in the general hypertensive population unacceptably low. This is due to the fact that EH is a highly heterogenous disease with a multifactorial and complex etiology, still not fully elucidated [5]. Recently, chronic inflammation triggered and sustained by excessive immune system activation has been implicated as a novel contributor to the pathogenesis of the disease [6]. Towards this direction, accumulating evidence has highlighted the role of toll-like receptors (TLRs) as major effectors of innate immunity, in promoting inflammation, oxidative stress, and endothelial dysfunction—all major pathophysiologic components mediating vascular damage in EH [7,8,9,10].

Taking into account the novelty of data in this emerging field, this review highlights the contribution of innate immunity in the pathogenesis of hypertension by clarifying the deleterious role of TLR signaling in promoting inflammation and facilitating hypertensive vascular damage.

## 2. Pathogenesis of Hypertension

It has been strongly supported that the pathogenesis of EH mainly consists of a noxious interplay between sophisticated neural, vascular, renal, and hormonal mechanisms, of which increased activation of the sympathetic nervous (SNS) and the renin-angiotensin system (RAS) prevail [5]. However, in recent years, it has been advocated that the core pathophysiologic event behind most of these mechanisms operating at the biological level is inflammation [11]. More specifically, a growing amount of evidence has demonstrated that multiple inflammatory mechanisms including pro-inflammatory cytokine and chemokine expression, cell infiltration and oxidative stress—all triggered and sustained by excessive immune system activation—are highly upregulated in the hypertensive environment [6,12].

## 3. The Role of the Immune System in the Pathogenesis of Hypertension

The immune system consists of both innate and adaptive immunity. Innate immunity represents the first line of defense of the human body and its main effector cells include dendritic cells, macrophages, granulocytes, natural killer (NK) cells, B cells, and mast cells, all of which act rapidly and non-specifically once activated by the presenting antigens. In contrast, adaptive immunity consists of T and B lymphocytes that mainly depend upon antigen recognition by the antigen-presenting cells in order to elicit a more robust inflammatory response [13].

**Figure 1:**
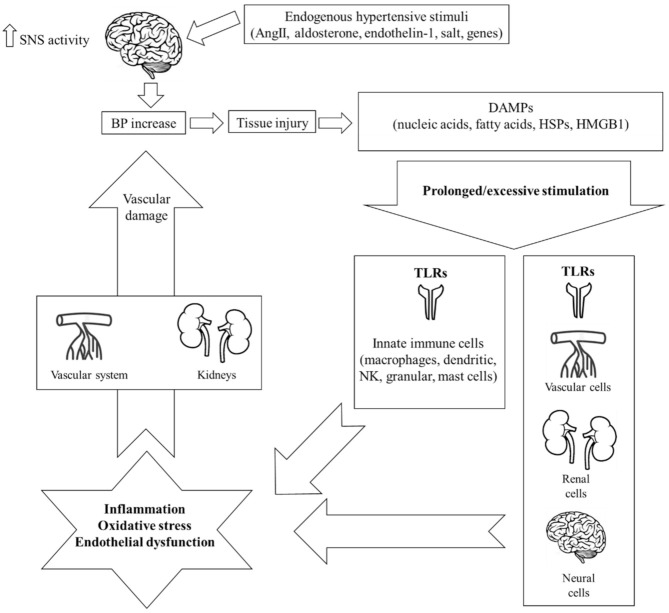
Schematic representation of the mechanisms of innate immunity implicated in the pathogenesis of hypertension. Endogenous stimuli including angiotensin II (AngII), aldosterone, endothelin-1, salt, and several genes initially increase central nervous signaling and thus sympathetic nervous system (SNS) activity, which, in turn, provokes a slight elevation in blood pressure (BP). This event leads to tissue injury and the release of endogenous intra- or extracellular molecules including cell-derived nucleic acids, fatty acids, heat shock proteins (HSPs), and high-mobility group box-1 (HMGB1), all termed damage-associated molecular patterns (DAMPs). In the hypertensive environment, an excessive or prolonged DAMP-mediated stimulation of innate immunity leads to an aberrant immunologic response through the activation of toll-like receptors (TLRs). The result is chronic low-grade inflammation, oxidative stress, and endothelial dysfunction, all of which inflict damage on the kidneys and the vascular system, thus further increasing BP. Importantly, increased TLR expression and subsequent activation has been documented not only on immune but also on non-immune cells of the central nervous, renal, and vascular system, all major contributors in the pathogenesis of hypertension.

Both systems are actively implicated in the pathogenesis of EH. More specifically, it has been hypothesized that hypertensive stimuli including angiotensin II (AngII), aldosterone, endothelin-1, salt, and several genes (i.e., ADRA2A [10q24-q26] (which is the predominant subtype gene modulating SNS outflow in the brain), ADRA2C [4p16.1], and ADRA2B [2p13-q13]) [14,15] increase central nervous signaling and thus SNS activity, which, in turn, provokes slight elevations in blood pressure (BP) [11]. This initial event leads to tissue injury and the release of endogenous intra- or extracellular molecules including cell-derived nucleic acids, fatty acids, heat shock proteins (HSPs), and high-mobility group box-1 (HMGB1), all termed damage-associated molecular patterns (DAMPs). Under normal conditions, DAMPs represent an acute “alarm signal”, warning the host to activate its defense mechanisms of which innate immunity is the first and most crucial one. In the hypertensive environment, though, it has been hypothesized that the pronounced DAMP-mediated stimulation of the immune system leads to significant inflammatory responses through two distinct pathways: i) the direct stimulation of innate immune cells further activating deleterious mechanisms including organ infiltration, chemokine and pro-inflammatory cytokine production, oxidative stress, phagocytosis, and complement activation, or ii) the consequent activation of adaptive immunity [6]. Activation of the adaptive immune response leads to the polarization of naive CD4+ T helper lymphocytes (T_h_0) towards pro-inflammatory T helper T_h_1 and T_h_17 phenotypes that produce reactive oxygen species (ROS), interferon (IFN)-γ, and interleukin (IL)-17 [16]. The net effect is a state of chronic low-grade inflammation that leads to additional vascular dysfunction, increased BP, tissue injury, and finally, the release of more DAMPs, which, in turn, flare up and maintain immune system hyperresponsiveness [17]. As a result, a vicious cycle of immune system activation and aberrant vascular inflammation is installed, ending up in target-organ damage and the further progression of EH [12] (Figure 1).

Whereas data support the participation of adaptive immunity in the pathogenesis of hypertension [16,18], the extent of the involvement of innate immunity, the precise mechanisms that stimulate it as well as its subsequent firing effects on adaptive immunity are still not well understood. To this end, the key role of TLRs in stimulating innate immunity and promoting inflammation in the hypertensive environment, merits further investigation.

## 4. Toll-Like Receptors

TLRs are type I transmembrane proteins belonging to the class of pattern recognition receptors (PRRs). In general, PRRs are in charge of recognizing and initiating an inflammatory reaction in response to unique, evolutionary conserved motifs termed either pathogen-associated molecular patterns (PAMPs), which are produced from viral and bacterial products, or DAMPs, which are released in the context of tissue damage, cellular stress, or cell death [10,19]. To date, 10 TLR subtypes have been identified in humans [TLR (1–10)] classified into two subfamilies according to their localization—the cell surface TLRs (1, 2, 4–6), expressed on the cell surface, and the intracellular TLRs (3, 7–10), localized in the endosomal compartment [10].

TLRs constitute the primary and most crucial step in the initiation of the inflammatory response by innate immunity. Upon activation by a specific PAMP or DAMP, TLRs fire an intracellular signal transduction cascade through two different pathways, namely (i) the myeloid differentiation primary response protein 88 (MyD88)-dependent pathway, which induces the activation of the early phase nuclear factor-κB (NF-κB), and (ii) the myD88-independent pathway (Toll/interleukin-1 receptor domain-containing adaptor protein inducing interferon-b [TRIF] dependent), which induces the activation of the late phase NF-κB. Both pathways culminate in the production and release of proinflammatory cytokines, chemokines, and several co-stimulatory factors all of which facilitate the inflammatory response [10,20,21]. In the hypertensive environment, TLRs are subject to excessive or prolonged DAMP-mediated stimulation that leads to an exaggerated innate immune inflammatory response and vascular damage. 

## 5. The Role of Toll-Like Receptors in the Pathogenesis of Hypertension

A concise amount of data has demonstrated evidence of increased TLR activation leading to inflammation in hypertension [8,9,10,22]. Importantly, enhanced TLR expression has been documented not only on immune cells but also on several non-immune cells across the renal, vascular, and neural tissues, representing the three most vital target organs involved in the pathogenesis of EH (Table 1).

### 5.1. Central Nervous System 

The crucial role of the central nervous system (CNS) in the pathogenesis of EH is mainly driven by the hypothalamic paraventricular nucleus (PVN). PVN is considered as one of the most important endocrine–autonomic control areas involved in the sympathetic regulation of BP, baroreflex function and body fluid homeostasis [37]. AngII, as part of the local RAS of the brain, exerts a potent inflammatory effect within the PVN by stimulating the production of various pro-inflammatory cytokines such as tumor necrosis factor (TNF)-α, interleukin (IL)-1β, and IL-6, and by promoting oxidative stress. The net result is a potent neuroinflammatory response strongly linked to an enhanced sympathetic output and increased BP [38,39]. 

TLR signaling pathways occupy a key role in this mechanistic crosstalk, taking into account that enhanced sympathoexitation and BP elevation have been shown as a result of TLR activation, mainly TLR4 [40] In fact, among all TLRs, TLR4 is constitutively and abundantly expressed in the CNS, and its role in promoting inflammation and vascular dysfunction has been well-demonstrated in several experimental models from normotension to hypertension [41,42]. In this context, Masson et al. showed that increased TLR4 expression in the PVN of normotensive rats was associated with acute autonomic dysfunction through mechanisms of microglial activation, neuronal inflammation, and endoplasmic reticulum stress. More specifically, acute TLR4 activation promptly increased the heart rate and plasma norepinephrine (NE) concentration, while simultaneously decreasing heart rate variability, cardiac vagal activity, and baroreflex sensitivity, suggesting that TLR4 firing can potentially decrease parasympathetic and increase sympathetic activity in the CNS [23]. Furthermore, in a model of spontaneously hypertensive rats (SHRs), Dange et al. found significantly higher levels of TLR4 in the PVN, which were associated with a potent inflammatory response, as evidenced by increased levels of HMGB1, augmented production of pro-inflammatory cytokines and inducible nitric oxide synthase (iNOS), and enhanced activity of NF-κB [25]. Importantly, the inflammatory response was abrogated upon treatment with an anti-TLR4 antibody, followed by significant reductions in the circulating plasma NE and mean BP (MBP) [25]. Similarly, in another model of SHRs, Li et al. found significantly upregulated levels of TLR4 bound with increased NF-κB activity and enhanced pro-inflammatory cytokine production in the PVN, all of which were abolished upon treatment with an AngII type 1 receptor antagonist, namely telmisartan. From a clinical point of view, treatment with telmisartan resulted in significant reductions of plasma NE and MBP, thus implying a beneficial profile of telmisartan in CNS, mediated through TLR4 signaling within the PVN [26]. 

The involvement of TLRs in the crosstalk between CNS inflammation and BP elevation has been also demonstrated in hypertensive models of exogenous AngII administration. More specifically, in a study by Ding et al., sustained AngII infusion into CNS caused a notable upregulation and activation of TLR4, followed by an excess immune-mediated inflammatory response including enhanced microglial activation and ROS production. Subsequently, sustained BP elevation was observed. Of interest, the overall effect was more pronounced in mice expressing the lectin-like oxidized low-density lipoprotein scavenger receptor-1 which has been linked to TLR4 activation, thereby reinforcing the observation that a TLR4 signaling pathway can mediate inflammatory responses in the brain linked to sympathoexcitation [27]. Likewise, in another study by Dange et al., AngII-induced hypertensive rats exhibited a dramatic upregulation of TLR4 within the PVN associated with a significant production of pro-inflammatory cytokines and a marked increase in plasma circulating NE and MBP. Furthermore, noticeable cardiac hypertrophy and impairment of cardiac diastolic function were observed. On the contrary, the blockade of TLR4 had a contrasting effect on AngII-mediated actions in favor of vasodilation, as indicated by significantly reduced plasma NE levels and MBP. In addition, delayed progression of target organ damage was observed, as evidenced by a reduction in cardiac hypertrophy and improvement of cardiac diastolic dysfunction [28]. 

Contrary to TLR4, for which most evidence exists, data concerning the involvement of other TLR signaling pathways in CNS is scarce. In a model of pre-hypertensive SHRs, the AngII- and nicotine-mediated stimulation of TLR7/8 and 9 resulted in an accentuated pro-inflammatory immune response, as evidenced by markedly increased levels of IL-6 and IL-1β, both in vitro and in vivo. However, in normotensive rats, nicotine showed a clearly contrasting effect by suppressing the TLR-mediated inflammatory response, whereas AngII had no effect at all [24]. Overall, it was concluded that the innate immune system is abnormally primed to be highly proinflammatory prior to the onset of high BP, and that its neurohormonal modulation could be implicated as a pathogenic mechanism in the development of hypertension.

### 5.2. Vascular System

Vascular inflammation and damage as a consequence of oxidative stress and endothelial dysfunction are a hallmark of EH and are already evident from the very early stages of the disease. Considering that TLRs are constitutively expressed on endothelial and vascular smooth muscle cells of different vascular beds, it has been supported that TLR signaling pathways are strongly involved in this pathophysiological process [43]. 

Towards this direction, a concise amount of data has demonstrated that TLR4 exerts a highly inflammatory effect by inhibiting the activity of antioxidant enzymes, activating nicotinamide adenine dinucleotide phosphate (NADPH) oxidase and upregulating the production of pro-inflammatory cytokines. By facilitating these actions, altogether, TLR4 promotes endothelial dysfunction, smooth muscle cell proliferation, and subsequently, vascular damage [44,45,46]. On the contrary, TLR4 loss-of-function exerts a protective effect on vascular integrity due to the downregulation of inflammatory cytokines and reduced ROS production and atherosclerosis [47,48,49]. Furthermore, TLR2 activity has been associated with vascular dysfunction through the inhibition of endothelial nitric oxide (NO) bioavailability and enhanced ROS production [50,51]. In addition, TLR9 has recently emerged as a novel contributor to vascular inflammation through unique proinflammatory and pro-oxidative signaling pathways [22,52].

The role of TLRs as potential mediators linking inflammation with vascular damage and BP elevation has been well-demonstrated in several experimental models from normotension to hypertension. Within the vasculature of normotensive rats, short-term treatment with a TLR9 agonist significantly increased ROS production and reduced NO bioavailability. As a result, the mesenteric resistance arteries were made less sensitive to vasodilatation induced by Ach and more prone to contraction induced by NE, whereas a significant increase in systolic blood pressure (SBP) was observed. Similarly, in SHRs, treatment with a TLR9 antagonist significantly lowered SBP, an effect that was sustained throughout the whole treatment period but returned to pre-treatment values soon after treatment discontinuation [29]. In another SHR model, it was demonstrated that increased TLR4 activation, as mediated by AngII, significantly upregulated NADPH oxidase activity, thereby stimulating ROS production and promoting oxidative stress. In contrast, TLR4 inhibition completely abolished those deleterious effects, whereas, at the same time, it improved vasodilatory responses to Ach, reduced vasoconstrictor responses to phenylephrine, and resulted in significant reductions in SBP, diastolic BP (DBP), MBP, and heart rate [30]. Correspondingly, in another SHR model, TLR4 inhibition decreased ROS generation, thereby eliciting a blunted vascular contractile response to NE followed by a significant reduction in MBP [31,32]. 

Further evidence reinforcing the contribution of TLRs to the pathogenesis of hypertensive vascular damage stems from AngII-induced hypertension models. In this context, it was shown that the AngII-induced upregulation of TLR4 leads to the increased production of inflammatory mediators and ROS, closely linked to considerable vascular dysfunction. More specifically, TLR4 activation resulted in significant reductions in the lumen diameter of small mesenteric arteries and an increase in the wall thickness and wall to lumen ratio, all findings being indicative of vascular remodeling. In addition, decreased numbers of smooth muscle and endothelial cells along with reduced distensibility and enhanced collagen deposition were observed, thus highlighting a condition of increased vascular stiffness. From a functional point of view, arterial segments exposed to phenylephrine showed enhanced contractile responses linked with increments in SBP. On the contrary, anti-TLR4 treatment had a totally opposing effect by improving all the detrimental effects of TLR activation, thereby confirming that AngII-mediated TLR4 activation strongly participates in the pathogenesis of hypertension by affecting various structural, mechanical, and functional properties of the vasculature [33]. Finally, in a model of NG-nitro-L-arginine methyl ester (L-NAME)-induced hypertension, inhibition of TLR4 signaling was associated with reduced contractility and enhanced vasodilation in isolated mesenteric arteries [34].

Overall, the aforementioned data indicate that TLRs are potent inducers of endothelial dysfunction and oxidative stress in the vasculature and therefore possess a substantial role in the development of hypertensive vascular damage. 

### 5.3. Renal System

The kidneys hold a fundamental role in the pathogenesis of hypertension by handling one of the most crucial mechanisms in charge of regulating fluid balance and BP—the RAS. Recently, an increasing amount of data has shown that RAS exerts a significant immune-mediated renal inflammatory effect, mainly driven by its prime component, AngII [41]. In fact, AngII has been strongly implied as a very potent proinflammatory mediator stimulating several immunological effector pathways, the net result of which is the development of renal injury and further progression of hypertensive renal damage [53,54]. One of those critical pathways through which AngII exerts its pro-inflammatory properties is increased activation of NF-kB which is a common denominator of the TLR signaling cascade, thus implying a role for TLRs in renal inflammatory damage. 

TLRs are constitutively expressed on renal epithelial, mesangial, and tubular cells [55]. In addition, it has been demonstrated that TLR4 expression is highly upregulated by AngII in several models of kidney disease [56,57]. Subsequent activation of TLR4 contributes substantially to renal inflammation and fibrosis, as evidenced by enhanced NF-kB production and chemokine expression [58,59,60].

Despite the evidence linking TLRs with renal damage, nevertheless, their role as an upstream signaling mechanism particularly in hypertension is not fully elucidated yet and existing data is scarce. Recently, in a mouse model of AngII-induced hypertension, TLR4-deficient mice were protected from the AngII-induced renal injury by a robust antioxidant mechanism which was associated with attenuated pro-inflammatory cytokine production, reduced macrophage activation, and decreased fibrosis. In the same model, TLR4 deficiency was associated with a blunted response to AngII-induced increases in BP and an amelioration of several renal vascular indices including intra-renal vascular resistance and renal cortical blood flow [35]. Finally, in a model of aldosterone-induced hypertension, TLR4 activation was associated with the downstream release of several inflammatory mediators leading to tubulointerstitial damage and fibrosis, whereas anti-TLR4 treatment significantly reversed those deleterious effects [36].

## 6. Conclusions

Although the etiology of EH is multifactorial and complex, chronic low-grade inflammation, triggered and sustained by excessive immune system activation, partly mediated through TLRs signaling pathways has emerged as a novel key player in the pathogenesis of the disease. Towards this direction, accumulating evidence demonstrates that TLRs significantly promote inflammation and are potent inducers of endothelial dysfunction and oxidative stress, which form the major pathophysiologic background behind vascular damage in EH. In addition, several data confirm that the activation of TLRs affects several structural, mechanical, and functional properties of the vasculature contributing to hypertensive vascular damage, while the above effects are reversed with specific anti-TLR treatment. Finally, evidence of enhanced TLR activity in hypertension has been documented not only on immune but also on several non-immune cells across the renal, vascular, and neural tissue that form the pathogenetic core systems operating in hypertensive disease. To this end, a plausible biological and clinical association linking TLRs, immune-driven inflammation, and vascular damage is evidently apparent in EH, therefore highlighting the crucial role of TLRs in the pathogenesis of the disease as well as their potential therapeutic value.

## Figures and Tables

**Table 1:** Experimental populations where TLR activation has been documented and the relevant subtypes of TLRs involved in the pathogenesis of hypertension according to organ system.

Organ System	TLR Subtype	Experimental Populations
Central Nervous System	TLR4 TLR7 TLR8 TLR9	Normotension [23] Pre-hypertensive SHRs [24] SHRs [25,26] AngII-induced hypertension [27,28]
Vascular System	TLR2 TLR4 TLR9	Normotension [29] SHRs [29,30,31,32] AngII-induced hypertension [33] L-NAME induced hypertension [34]
Renal System	TLR4	AngII-induced hypertension [35] Aldosterone-induced hypertension [36]

AngII: angiotensin II; L-NAME: NG-nitro-L-arginine methyl ester; SHRs: spontaneously hypertensive rats; TLR: toll-like receptor.

## Data Availability

Not applicable.
